# Hypocholesterolemic and Antiatherosclerotic Potential of* Basella alba* Leaf Extract in Hypercholesterolemia-Induced Rabbits

**DOI:** 10.1155/2015/751714

**Published:** 2015-11-30

**Authors:** Gunasekaran Baskaran, Shamala Salvamani, Azrina Azlan, Siti Aqlima Ahmad, Swee Keong Yeap, Mohd Yunus Shukor

**Affiliations:** ^1^Department of Biochemistry, Faculty of Biotechnology and Biomolecular Sciences, Universiti Putra Malaysia (UPM), 43400 Serdang, Selangor, Malaysia; ^2^Department of Nutrition and Dietetics, Faculty of Medicine and Health Sciences, Universiti Putra Malaysia (UPM), 43400 Serdang, Selangor, Malaysia; ^3^Institute of Bioscience, Universiti Putra Malaysia (UPM), 43400 Serdang, Selangor, Malaysia

## Abstract

Hypercholesterolemia is the major risk factor that leads to atherosclerosis. Nowadays, alternative treatment using medicinal plants gained much attention since the usage of statins leads to adverse health effects, especially liver and muscle toxicity. This study was designed to investigate the hypocholesterolemic and antiatherosclerotic effects of* Basella alba* (*B. alba*) using hypercholesterolemia-induced rabbits. Twenty New Zealand white rabbits were divided into 5 groups and fed with varying diets: normal diet, 2% high cholesterol diet (HCD), 2% HCD + 10 mg/kg simvastatin, 2% HCD + 100 mg/kg* B. alba* extract, and 2% HCD + 200 mg/kg* B. alba* extract, respectively. The treatment with* B. alba* extract significantly lowered the levels of total cholesterol, LDL, and triglycerides and increased HDL and antioxidant enzymes (SOD and GPx) levels. The elevated levels of liver enzymes (AST and ALT) and creatine kinase were noted in hypercholesterolemic and statin treated groups indicating liver and muscle injuries. Treatment with* B. alba* extract also significantly suppressed the aortic plaque formation and reduced the intima: media ratio as observed in simvastatin-treated group. This is the first* in vivo* study on* B. alba *that suggests its potential as an alternative therapeutic agent for hypercholesterolemia and atherosclerosis.

## 1. Introduction

Hypercholesterolemia is closely associated with atherosclerosis, which is the principal cause of mortality in world population. Hypercholesterolemia is characterized by increased serum concentrations of low-density lipoprotein (LDL) and triglycerides (TG) [1]. Accumulation of oxidized LDL leads to atherosclerotic plaque formation which contributes to stroke, myocardial infarction, and cardiovascular diseases (CVDs) [2]. It is well known that the hypocholesterolemic drugs are effective in lowering LDL but the long term consumption causes adverse effects such as liver and muscle injuries, rhabdomyolysis, myopathy, and acute renal failure. Thus, the investigation and usage of natural products from plant origin in treating various diseases including CVDs have gained much attention [3, 4].

The potential of medicinal plants that exhibit hypocholesterolemic and antiartherosclerotic effects is still largely unexplored and could be an effective and safe alternative strategy for the treatment of hypercholesterolemia. In previous study, we have screened the HMG CoA reductase inhibitory activity of 25 medicinal plants extracts.* Basella alba *(*B. alba*) extract showed the highest enzyme inhibition, about 74% [5].

*B. alba* is known as Indian spinach and Remayung locally and belongs to the family of Basellaceae.* B. alba *is a wildly cultivated vegetable that has been used from ancient time due to its various pharmacological activities such as antifungal, antiulcer, anticonvulsant, antihypertensive, and many more activities [6]. In Asian countries, the stem and leaf of* B. alba *have been employed as traditional medicine to treat dysentery, skin diseases, hemorrhages, anemia, constipation, gonorrhea, and cancer [7–9].

There are no* in vivo* reports on the effects of* B. alba* on hypercholesterolemia up to date. Therefore, the present study was aimed at investigating the hypocholesterolemic and antiatherosclerotic properties of* B. alba* in hypercholesterolemic rabbits and also at determining the antioxidant capacity of this extract.

## 2. Materials and Methods

### 2.1. Preparation of* B. alba* Methanol Extract

*B. alba* leaf was purchased from a local market in Seri Kembangan, Selangor, Malaysia. A voucher specimen was deposited in the Institute of Bioscience, Universiti Putra Malaysia (voucher number SK 2087/12).* B. alba* leaf was washed thoroughly and air-dried at room temperature for overnight. The leaf was grounded using a blender (MX 8967, Panasonic) and subjected to methanol 50% (v/v) distillation for 48 hours. After filtration, the leaf extract was isolated using a separatory funnel. The crude methanolic extract of* B. alba* was concentrated using rotary evaporator (Heidolph) under reduced pressure at 40°C and freeze-dried at −40°C for further analysis.

### 2.2. Animals and Experimental Design

Twenty male New Zealand white rabbits weighing 1.5–1.8 kg were purchased from local supplier. The animal studies were performed according to guidelines approved by the Institutional Animal Care and Use Committee (IACUC) of Universiti Putra Malaysia (UPM/IACUC/AUP-R011/2013). The rabbits were placed individually in stainless steel cages and were fed standard rabbit pellets for 1 week for acclimatization. Throughout the study, all the rabbits were kept in a 12 h light-dark cycle room with almost constant temperature at 23–25°C.

The* in vivo* study was carried out for 12 weeks. The rabbits were randomly divided into 5 groups (*n* = 4): Group 1: control rabbits fed with standard diet for 12 weeks; Group 2: rabbits fed with 2% high cholesterol diet (HCD) for 12 weeks; Group 3: rabbits fed with 2% HCD for 8 weeks and treatment with simvastatin (10 mg/kg) for 4 weeks; Group 4: rabbits fed with 2% HCD for 8 weeks and treatment with* B. alba* extract (100 mg/kg) for 4 weeks; and Group 5: rabbits fed with 2% HCD for 8 weeks and treatment with* B. alba* extract (200 mg/kg) for 4 weeks.

The high cholesterol diet was prepared by dissolving 2% cholesterol (USP grade, anhydrous; Sigma Chemical Co., Missouri, USA) in 99% chloroform and sprayed on standard pellets. Butylated hydroxyanisole (0.02% of diet) was dissolved in chloroform to reduce oxidation of cholesterol. The chloroform was evaporated by exposing the diets in well-ventilated fume hoods at room temperature for overnight. The diets were vacuum-packed and stored in −20°C freezer. All the rabbits received about 150 g pellets per day, with or without cholesterol supplementation, and water was provided* ad libitum*. Food and water consumption were recorded daily, while the body weight was measured every 2 weeks. Blood samples were collected at 0, 4, 8, and 12th week via ear marginal vein using 23-gauge butterfly needle and 3 mL syringes into EDTA and heparinised tubes. At the end of the study, the rabbits were euthanized with overdose of sodium pentobarbital through intravenous injection.

### 2.3. Measurement of Serum Lipids

Serum total cholesterol (TC), LDL, HDL, and triglycerides (TG) levels were determined using Roche kit (Penzberg, Germany) and measured spectrophotometrically using Hitachi chemistry analyzer (Tokyo, Japan).

### 2.4. Liver and Muscle Test

The serum levels of ALT, AST, and creatine kinase (CK) were evaluated by enzymatic kit (Randox Laboratories, Crumlin, UK) using Hitachi chemistry analyzer (Tokyo, Japan).

### 2.5. Antioxidant Activities

Superoxide Dismutase (SOD) activity was measured by RANSOD kit (Randox Laboratories, Crumlin, UK) using Vitalab Selectra Analyzer (Merck, Darmstadt, Germany). The collected erythrocytes were washed four times with NaCl solution (0.9%, 3 mL) by centrifugation at 1000 ×g for 10 min. Cold distilled water was added up to 2 mL to the packed erythrocytes, vortexed for 10 s, and incubated at 4°C for 15 min. The lysate was then diluted with phosphate buffer (pH 7, 0.01 mol/L) and mixed thoroughly. The absorbance of the mixture was determined at 505 nm. The Glutathione Peroxidase (GPx) activity was measured by diluting 0.05 mL serum with 2 mL of RANSEL kit diluting agent (Randox Laboratories, Crumlin, UK) and the mixture was read at 340 nm using Vitalab Selectra Analyzer (Merck, Darmstadt, Germany).

### 2.6. Histological Analysis

The aortic arch of the rabbits was removed, cleaned, dried, and fixed in 10% neutral buffer formalin. The tissues were embedded in paraffin, cut in 5 *μ*m sections, and stained with hematoxylin and eosin. The atherosclerotic lesion was analyzed for the thickness of intima, media, and intima: media ratio of 4 rabbits per group under a light microscope equipped with image analyzer system (Olympus, Germany).

### 2.7. Statistical Analysis

The data obtained are expressed as mean ± SD. All groups were analyzed using SPSS program version 19.0. One-way analysis of variance (ANOVA) followed by Dunnett's post hoc test for multiple comparisons among the groups was performed. The difference between groups was considered to be statistically significant when *p* < 0.05.

## 3. Results

### 3.1. Effect of* B. alba* Extract on Body Weight

In Table 1, rabbits fed with 2% cholesterol diet for 12 weeks showed significant increase (*p* < 0.05) in body weight compared to the normal control. Treatment with simvastatin and* B. alba *(100 and 200 mg/kg) for 4 weeks managed to reduce the body weight compared to the untreated hypercholesterolemic rabbits.

### 3.2. Effect of* B. alba* Extract on Serum Lipid Profile

As shown in Table 2, serum levels of TC, LDL, and TG were significantly higher (*p* < 0.05) in rabbits fed with HCD compared to the normal diet group after 8 weeks. There were significant decreases (*p* < 0.05) in the level of TC, LDL, and TG at week 12, after 4 weeks of treatment with simvastatin and* B. alba* extract. Administration with 10 mg/kg of simvastatin, used as a positive control, significantly decreased 58.9, 51.1, and 40.9% in TC, LDL, and TG levels, respectively. Administration of* B. alba* at 100 and 200 mg/kg decreased TC level by 49 and 54.2%, respectively, LDL level by 45 and 50.1%, respectively, and TG level by 34.9 and 39.7%, respectively. The TC, LDL, and TG lowering effects of* B. alba* (200 mg/kg) were not significantly different with simvastatin.* B. alba *at dose of 200 mg/kg has significantly higher hypocholesterolemic effect than that of 100 mg/kg. Meanwhile, the HDL level of hypercholesterolemic control rabbits was significantly lower (*p* < 0.05) compared to the normal control and treatment groups at week 12. Treatment with simvastatin and* B. alba* (100 and 200 mg/kg) showed significant increase (*p* < 0.05) in HDL levels, 31.7, 39.6, and 53.4%, respectively.* B. alba* (200 mg/kg) increases the HDL level more effectively than simvastatin.

### 3.3. Evaluation of Liver and Muscle Injuries

The hypercholesterolemia-induced rabbits showed significant increase (*p* < 0.05) in ALT, AST, and CK levels as presented in Table 3. The results revealed that the treatment with* B. alba* extract (100 and 200 mg/kg) significantly decreased (*p* < 0.05) ALT (40.5 and 44.9%, resp.), AST (37.3 and 43.7%, resp.), and CK (24.2 and 22.8%, resp.) levels while the treatment with simvastatin (10 mg/kg) showed significant elevation (*p* < 0.05) in the levels of ALT (61.4%), AST (64.1%), and CK (34%).

### 3.4. Evaluation of Serum Antioxidant Levels

As shown in Table 4, the hypercholesterolemic control showed significant reduction (*p* < 0.05) in the levels of SOD and GPx throughout the study.* B. alba-*treated rabbits (100 and 200 mg/kg) caused significant increase (*p* < 0.05) in SOD by 5 and 5.4%, respectively, and GPx by 15 and 21%, respectively. Meanwhile, simvastatin-treated groups showed significant reduction (*p* < 0.05) in SOD and GPx, 4 and 19%, respectively.

### 3.5. Effect of* B. alba* Extract on Atherosclerotic Lesion

**Figure 1:**
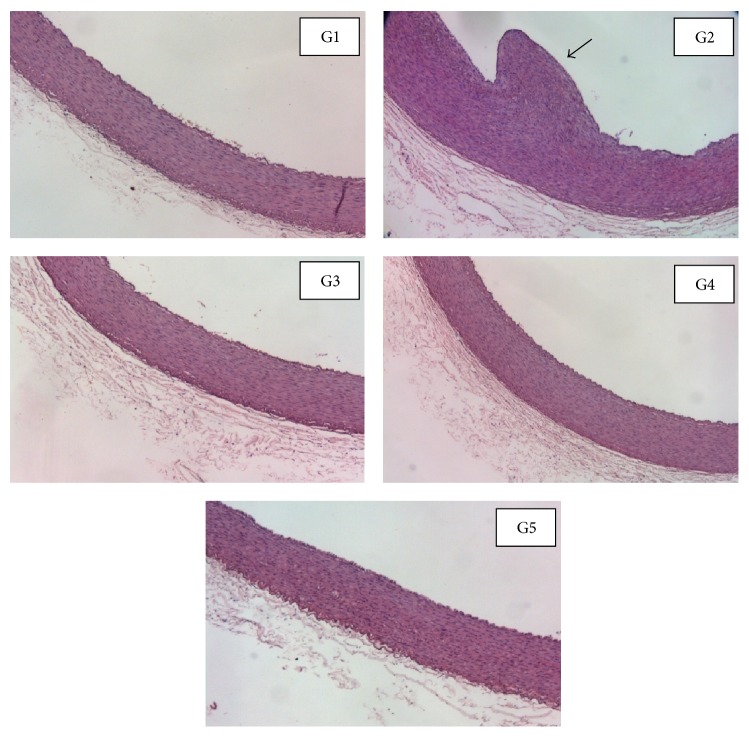
Representative photographs of rabbits' aortic arch from 5 groups stained with H&E. The aorta of a control hypercholesterolemic rabbit (G2) showing a large intimal plaque (arrow). G1: normal control, G2: hypercholesterolemic control, G3: simvastatin (10 mg/kg) treated, G4:* B. alba* extract (100 mg/kg) treated, and G5:* B. alba* extract (200 mg/kg) treated (Magnification 50x).

The atherosclerotic changes in aortic intimal surface of 5 groups are shown in Figure 1. Normal control group (G1) showed healthy aorta with uniform thickness and intact endothelial lining. On the other hand, hypercholesterolemic control group (G2) caused alteration in the aortic wall with the appearance of a large atheromatous plaque and demonstrated a remarkable intimal thickening of aorta. In contrast, treatment with simvastatin (G3) and* B. alba *(G4 and G5) revealed significant decrease in the thickening of intima and no plaques were detected in the aortic walls.  Table 5 summarizes the thickness of intima, media, and intima/media ratio of the 5 groups at week 12. The hypercholesterolemic control group showed significant difference (*p* < 0.05) with the highest value of intima and media thickness and intima/media ratio compared to other groups. On the other hand, significant reductions (*p* < 0.05) were noted in the thickness of intima and media as well as intima/media ratio of simvastatin and* B. alba* (100 and 200 mg/kg) treated groups compared to the hypercholesterolemic control group. There was no significant difference (*p* < 0.05) in the intima/media ratio between simvastatin and* B. alba* (200 mg/kg) treated groups.

## 4. Discussion

This is the first report that demonstrates the oral administration of* B. alba* extract in hypercholesterolemia-induced rabbits. Rabbit is a good model to study hypercholesterolemia and atherosclerosis since its lipoprotein profile and metabolism are more similar to humans than that of rat or mouse [10]. In the present study, simvastatin, a potent hypocholesterolemic drug, was used as a positive control because it has known mechanism of action in inhibiting HMG-CoA reductase [11].

HCD feeding showed significant elevation of TC, LDL, and TG, which increase lipid peroxidation and influence the development of atherosclerosis, in agreement with several studies [12]. In contrast, a significant decrease noted in serum HDL in hypercholesterolemic rabbits was also reported by Ismail et al. [4]. The rabbits administered simvastatin or* B. alba* extract had reduction in body weight and serum levels of TC, LDL, and TG and significant increase noted in HDL levels.

LDL cholesterol is a primary target of atherosclerosis risk-reduction therapy. Excess LDL is mostly deposited in arterial wall and becomes a main component of atherosclerotic plaque formation, while HCD feeding has been reported to reduce fatty acid oxidation, resulting in the increase of serum TG which is considered as another risk factor for CVDs [13]. In our study,* B. alba* extract (100 and 200 mg/kg) elicited beneficial effects by attenuating the level of cholesterol including LDL and TG of the treated rabbits.* B. alba* extract (200 mg/kg) reduces TC, LDL, and TG levels as effectively as simvastatin.

HDL plays an essential role in protecting the membranes against oxidative damage. HDL is involved in the uptake and transport of cholesterol to the liver through reverse cholesterol transport process [14]. Epidemiological and clinical studies have shown that low level of HDL plays a crucial role in the atherogenic process [15]; thus, therapeutic approach to increase the HDL level is widely encouraged [16]. Significant increase in HDL as shown in* B. alba-*treated rabbits is a desirable criterion for an ideal hypercholesterolemic agent since it reduces the atherosclerotic risk.

The liver is the primary organ that is responsible for maintaining the cholesterol homeostasis. The marker enzymes, ALT and AST, were evaluated to detect the liver damage while CK was used to diagnose the muscle injury. These enzymes were reported to leak into the blood circulation when their cell membranes were injured [17]. The levels of the enzymes were found elevated in hypercholesterolemic control and simvastatin-treated rabbits compared to the normal group. This suggests high concentration of cholesterol and the usage of simvastatin caused liver and muscle damage [18, 19], whereas administration of* B. alba *extract for 4 weeks reduced the elevations of ALT, AST, and CK indicating its hepatic and muscle-protective effects.

Antioxidant enzymes (SOD and GPx) play essential roles in maintaining the physiological concentrations of oxygen and hydrogen peroxide by improving the dismutation of oxygen radicals [20]. A decrease in the level of SOD and GPx was observed in simvastatin-treated and hypercholesterolemic control groups. A significant decrease in GPx activity in simvastatin-treated group was also noted by Trocha et al. [21]; this could be due to the reduced antioxidant capacity in the serum of the animal model.

High cholesterol diet alters the* in vivo* antioxidant status in blood by increasing the oxygen free radicals that cause lipid peroxidation [22]. In our present study, feeding hypercholesterolemic diet for 8 weeks leads to the reduction in the activities of SOD and GPx. Many reports have also proved that hypercholesterolemia diminishes the activity of SOD [23, 24] and GPx [25]. The reduced level of SOD and GPx activities is associated with increased risk of CVD [26, 27]. The data obtained in this study suggested that* B. alba* extract is capable of enhancing the activity of SOD and GPx in hypercholesterolemia-induced rabbits; the effect could be due to the presence of phenolic compounds. Indeed, plant polyphenols have been reported to regulate antioxidative status by ameliorating the activity of antioxidant enzymes [20]. Therefore, this suggests that* B. alba* extract is capable of improving the antioxidant status and could be beneficial in managing oxidative damage and preventing lipid peroxidation.

Oxidized LDL molecules are commonly found in subendothelial layers. The accumulation of oxidized LDL in macrophages can stimulate proliferation of monocytes, smooth muscle cells, and endothelial cells. When the scavenging receptor for oxidized LDL on macrophages is upregulated, it leads to foam cells formation which are the major component of fatty streaks. This contributes to atheromatous plaque formation and thickening of intimal layer [28, 29]. The histopathological examination of aorta correlates with the serum biochemical data. The level of hypercholesterolemia was directly proportional with the severity of atherosclerotic plaque as observed in the aorta of hypercholesterolemic control group. Simvastatin and* B. alba-*treated rabbits revealed a significant reduction in aortic plaque and intimal thickening. In general,* B. alba* treatment (200 mg/kg) showed no significant difference in intima/media ratio compared to simvastatin-treated group. This suggests that* B. alba* extract (200 mg/kg) is as effective as simvastatin in treating atherosclerosis.

The mechanism by which* B. alba *inhibits the atherosclerotic plaque is not known but may be due to its antioxidant, hypocholesterolemic, and antiatherosclerotic effects such as decreasing oxidative stress, lowering the LDL level, reducing inflammation, and inhibiting macrophage accumulation. From the results obtained, it can be concluded that* B. alba* possesses therapeutic effects in treating hypercholesterolemia and atherosclerosis.

## 5. Conclusion

*B. alba* leaf extract could be an effective alternative treatment for hypercholesterolemia and atherosclerosis. The results from the present study showed that* B. alba* extract (200 mg/kg) effectively reduces the levels of TC, LDL, and TG and raised the level of HDL and antioxidants enzymes.* B. alba* leaf extract did not cause liver and muscle damage indicating that it is safe for consumption.* B. alba* successfully inhibited the atherosclerotic plaque formation in hypercholesterolemia-induced model. The finding from* in vivo* study is in good agreement with that of* in vitro *study with HMG CoA reductase, confirming the cholesterol lowering effect of* B. alba*. Further investigations are needed on the mechanisms of* B. alba* in inhibiting the atherosclerotic plaque formation. Isolation and identification of bioactive compounds of* B. alba* that are responsible for the observed effects are needed, which can be developed as a prophylactic agent against hypercholesterolemia and atherosclerosis.

## Figures and Tables

**Table 1:** Changes on body weight of rabbits between different groups.

Group	Body weight (kg)		
	Initial	Final	Change
G1	1.61 ± 0.16	2.22 ± 0.09	0.61 ± 0.13
G2	1.65 ± 0.17	2.55 ± 0.13	0.90 ± 0.06^*∗*^
G3	1.63 ± 0.10	2.31 ± 0.14	0.68 ± 0.08
G4	1.75 ± 0.15	2.48 ± 0.13	0.73 ± 0.10
G5	1.84 ± 0.06	2.54 ± 0.16	0.70 ± 0.19

G1: normal control, G2: hypercholesterolemic control, G3: simvastatin-treated (10 mg/kg), G4: *B. alba* extract-treated (100 mg/kg), and G5: *B. alba* extract-treated (200 mg/kg). All data are presented as the mean ± SD (*n* = 4 for each group). ^*∗*^Significantly different from others (*p* < 0.05).

**Table 2:** Levels of total cholesterol, LDL, triglycerides, and HDL in serum of rabbits from various groups.

	G1	G2	G3	G4	G5
Total cholesterol (mg/dL)					
Baseline	39.45 ± 2.95^a^	39.33 ± 3.38^a^	44.37 ± 3.87^a^	41.41 ± 4.58^a^	42.30 ± 4.33^a^
Week 4	42.66 ± 2.67^a^	472.65 ± 4.54^b^	480.83 ± 5.18^b^	506.92 ± 9.57^b^	467.08 ± 8.35^b^
Week 8	46.14 ± 4.06^a^	1104.98 ± 33.32^c^	1112.70 ± 28.33^c^	1002.53 ± 11.51^b^	1038.85 ± 12.35^b^
Week 12	53.57 ± 2.70^a^	1326.91 ± 19.47^d^	456.56 ± 5.23^b^	510.99 ± 8.14^c^	475.12 ± 10.94^b^
LDL level (mg/dL)					
Baseline	26.48 ± 0.72^a^	31.19 ± 0.97^a^	25.39 ± 0.43^a^	29.02 ± 1.13^a^	27.03 ± 0.82^a^
Week 4	33.92 ± 0.55^a^	426.15 ± 4.52^b^	440.66 ± 3.39^b^	421.68 ± 3.95^b^	435.94 ± 2.57^b^
Week 8	38.79 ± 0.77^a^	1157.09 ± 13.85^d^	1056.66 ± 15.38^c^	1001.23 ± 12.40^b^	1081.04 ± 4.06^c^
Week 12	45.96 ± 1.01^a^	1259.60 ± 15.43^d^	515.83 ± 14.03^b^	547.39 ± 12.81^c^	539.27 ± 13.05^b^
Triglyceride level (mg/dL)					
Baseline	110.13 ± 4.55^a^	116.46 ± 3.68^b^	106.88 ± 6.28^a^	114.39 ± 5.36^b^	107.56 ± 7.58^a^
Week 4	132.83 ± 7.84^a^	340.63 ± 8.74^c^	331.84 ± 4.97^b^	349.42 ± 3.41^b^	329.74 ± 6.95^b^
Week 8	156.38 ± 8.80^a^	654.58 ± 15.16^b^	650.96 ± 18.35^b^	639.52 ± 16.04^b^	661.12 ± 18.81^b^
Week 12	166.03 ± 5.81^c^	869.57 ± 15.38^d^	384.30 ± 14.63^a^	416.14 ± 16.73^b^	398.53 ± 10.66^a^
HDL level (mg/dL)					
Baseline	45.65 ± 0.758^b^	41.53 ± 5.27^a,b^	38.27 ± 0.71^a^	46.02 ± 1.15^b^	39.83 ± 5.30^a^
Week 4	46.94 ± 1.09^b,c^	38.13 ± 5.28^a,b^	37.30 ± 0.78^a^	44.31 ± 1.10^c^	37.09 ± 5.27^a^
Week 8	47.45 ± 1.15^c^	34.33 ± 5.18^a^	36.37 ± 2.66^a,b^	38.51 ± 3.35^b^	35.88 ± 3.92^a^
Week 12	47.25 ± 0.45^c^	28.27 ± 7.21^a^	47.93 ± 2.51^c^	53.78 ± 3.45^b^	55.05 ± 1.26^b^

G1: normal control, G2: hypercholesterolemic control, G3: simvastatin-treated (10 mg/kg), G4: *B. alba* extract-treated (100 mg/kg), and G5: *B. alba* extract-treated (200 mg/kg). All data are presented as the mean ± SD (*n* = 4 for each group). One-way ANOVA was performed followed by Dunnett's *post hoc* test for multiple comparisons. Within a week, values sharing the same superscript letters are not significantly different from each other (*p* < 0.05).

**Table 3:** Levels of ALT, AST, and CK in serum of rabbits from various groups.

	G1	G2	G3	G4	G5
ALT (U/L)					
Baseline	21.22 ± 1.27^a^	20.30 ± 1.47^b^	23.20 ± 1.37^b^	21.43 ± 1.53^b^	22.45 ± 0.75^b^
Week 4	21.05 ± 2.79^a^	43.10 ± 4.67^b^	47.75 ± 1.79^c^	44.85 ± 1.44^b,c^	46.18 ± 0.51^^c^^
Week 8	24.62 ± 1.65^a^	63.75 ± 3.48^b^	64.98 ± 6.99^b^	67.85 ± 1.36^b^	69.75 ± 1.48^b^
Week 12	29.15 ± 1.07^a^	91.80 ± 4.45^c^	104.85 ± 4.53^d^	40.35 ± 2.24^b^	38.43 ± 2.28^b^
AST (U/L)					
Baseline	29.65 ± 1.23^b^	29.65 ± 4.06^b^	28.18 ± 3.86^b^	25.93 ± 3.15^a^	27.13 ± 4.16^a,b^
Week 4	32.97 ± 3.78^a^	49.75 ± 8.63^b^	51.78 ± 6.57^b,c^	47.68 ± 5.57^b^	46.33 ± 1.33^b^
Week 8	36.97 ± 1.09^a^	67.48 ± 8.76^b^	69.30 ± 8.17^b^	67.65 ± 5.64^b^	71.65 ± 2.84^b^
Week 12	39.87 ± 2.37^a^	95.77 ± 8.54^c^	113.75 ± 4.46^d^	42.38 ± 3.46^b^	40.32 ± 4.00^b^
CK (U/L)					
Baseline	540.30 ± 22.85^a^	578.80 ± 20.72^a^	556.23 ± 51.60^a^	544.08 ± 35.39^a^	569.30 ± 36.96^a^
Week 4	555.50 ± 25.17^a^	900.48 ± 75.95^b^	886.55 ± 113.73^b^	865.90 ± 88.06^b^	881.45 ± 6.00^b^
Week 8	579.02 ± 22.00^a^	1466.00 ± 233.42^b^	1562.13 ± 196.69^b^	1325.28 ± 97.91^b^	1362.88 ± 104.08^b^
Week 12	597.80 ± 13.49^a^	1931.48 ± 223.55^c^	2093.95 ± 272.50^c^	1004.30 ± 100.27^b^	1051.03 ± 99.88^b^

G1: normal control, G2: hypercholesterolemic control, G3: simvastatin-treated (10 mg/kg), G4: *B. alba* extract-treated (100 mg/kg), and G5: *B. alba* extract-treated (200 mg/kg). All data are presented as the mean ± SD (*n* = 4 for each group). One-way ANOVA was performed followed by Dunnett's *post hoc* test for multiple comparisons. Within a week, values sharing the same superscript letters are not significantly different from each other (*p* < 0.05).

**Table 4:** Levels of antioxidant enzymes in serum of rabbits from various groups.

	G1	G2	G3	G4	G5
SOD (U/mL)					
Baseline	5.88 ± 0.17^a,b^	5.63 ± 0.25^a^	5.93 ± 0.11^b^	5.72 ± 0.15^a,b^	5.84 ± 0.12^a,b^
Week 4	5.98 ± 0.21^c^	5.49 ± 0.34^b^	5.61 ± 0.18^b^	5.39 ± 0.30^a^	5.33 ± 0.21^a^
Week 8	6.05 ± 0.22^c^	5.35 ± 0.29^b^	5.25 ± 0.25^b^	4.96 ± 0.17^a^	5.10 ± 0.31^a^
Week 12	6.20 ± 0.18^c^	4.91 ± 0.20^a^	5.04 ± 0.28^a^	5.21 ± 0.73^b^	5.38 ± 0.35^b^
GPx (U/L)					
Baseline	1243.73 ± 91.21^a^	1211.65 ± 101.19^a^	1443.10 ± 55.78^b^	1313.95 ± 232.95^b^	1401.20 ± 171.33^b^
Week 4	1330.85 ± 77.53^c^	1198.58 ± 90.38^a^	1351.83 ± 128.17^c^	1277.18 ± 184.68^b^	1274.78 ± 124.44^b^
Week 8	1459.45 ± 175.80^b^	1055.30 ± 62.23^a^	1205.00 ± 82.15^a^	1079.55 ± 213.70^a^	1169.20 ± 82.50^a^
Week 12	1595.35 ± 219.20^c^	885.23 ± 43.84^a^	975.53 ± 62.41^a^	1241.63 ± 181.98^b^	1418.5 ± 82.55^b,c^

G1: normal control, G2: hypercholesterolemic control, G3: simvastatin-treated (10 mg/kg), G4: *B. alba* extract-treated (100 mg/kg), and G5: *B. alba* extract-treated (200 mg/kg). All data are presented as the mean ± SD (*n* = 4 for each group). One-way ANOVA was performed followed by Dunnett's *post hoc* test for multiple comparisons. Within a week, values sharing the same superscript letters are not significantly different from each other (*p* < 0.05).

**Table 5:** Thickness of intima, media, and intima/media ratio of experimental rabbits at week 12.

Groups	Intima thickness (*µ*m)	Media thickness (*µ*m)	Intima/media
G1	1045.439 ± 80.26^a^	3350.768 ± 213.40^a^	0.312 ± 0.039^a^
G2	4203.296 ± 160.96^d^	6004.708 ± 102.28^d^	0.700 ± 0.023^d^
G3	1585.270 ± 111.40^b^	3809.198 ± 160.44^b^	0.416 ± 0.035^b^
G4	1786.301 ± 104.59^c^	3978.398 ± 85.94^b,c^	0.449 ± 0.019^c^
G5	1720.678 ± 152.25^b^	4058.203 ± 181.25^c^	0.424 ± 0.033^b^

G1: normal control, G2: hypercholesterolemic control, G3: simvastatin-treated (10 mg/kg), G4: *B. alba* extract-treated (100 mg/kg), and G5: *B. alba* extract-treated (200 mg/kg). All data are presented as the mean ± SD (*n* = 4 for each group). One-way ANOVA was performed followed by Dunnett's *post hoc* test for multiple comparisons. Within a column, values sharing the same superscript letters are not significantly different from each other (*p* < 0.05).
